# Diabetes Prevention in Adolescents: Co-design Study Using Human-Centered Design Methodologies

**DOI:** 10.2196/18245

**Published:** 2021-02-24

**Authors:** Julie M Pike, Courtney M Moore, Lisa G Yazel, Dustin O Lynch, Kathryn M Haberlin-Pittz, Sarah E Wiehe, Tamara S Hannon

**Affiliations:** 1 Pediatric and Adolescent Comparative Effectiveness Research Department of Pediatrics Indiana University School of Medicine Indianapolis, IN United States; 2 Division of Pediatric Endocrinology and Diabetology Department of Pediatrics Indiana University School of Medicine Indianapolis, IN United States; 3 Research Jam Indiana Clinical and Translational Sciences Institute Patient Engagement Core Indiana University Indianapolis, IN United States; 4 Children's Health Services Research Center Department of Pediatrics Indiana University School of Medicine Indianapolis, IN United States

**Keywords:** diabetes prevention, adolescents, human-centered design

## Abstract

**Background:**

The rise in pediatric obesity and its accompanying condition, type 2 diabetes (T2D), is a serious public health concern. T2D in adolescents is associated with poor health outcomes and decreased life expectancy. Effective diabetes prevention strategies for high-risk adolescents and their families are urgently needed.

**Objective:**

The aim of this study was to co-design a diabetes prevention program for adolescents by using human-centered design methodologies.

**Methods:**

We partnered with at-risk adolescents, parents, and professionals with expertise in diabetes prevention or those working with adolescents to conduct a series of human-centered design research sessions to co-design a diabetes prevention intervention for youth and their families. In order to do so, we needed to (1) better understand environmental factors that inhibit/promote recommended lifestyle changes to decrease T2D risk, (2) elucidate desired program characteristics, and (3) explore improved activation in diabetes prevention programs.

**Results:**

Financial resources, limited access to healthy foods, safe places for physical activity, and competing priorities pose barriers to adopting lifestyle changes. Adolescents and their parents desire interactive, hands-on learning experiences that incorporate a sense of fun, play, and community in diabetes prevention programs.

**Conclusions:**

The findings of this study highlight important insights of 3 specific stakeholder groups regarding diabetes prevention and lifestyle changes. The findings of this study demonstrate that, with appropriate methods and facilitation, adolescents, parents, and professionals can be empowered to co-design diabetes prevention programs.

## Introduction

Excess weight and obesity in youth continue to be a serious public health concern [1] and put youth at risk of developing type 2 diabetes (T2D) [2]. T2D in youth increased by 4.8% per year from 2002 to 2012 [3] and is predicted to increase fourfold by 2050 [4]. Minority youths are disproportionately affected [5,6]. Non-Hispanic Black youth experienced the largest annual increases (6.3%) in T2D compared to non-Hispanic White youth (0.6%) [3]. Furthermore, early onset of T2D and poor glycemic control increase the risk of diabetes-related complications and decrease life expectancy [7]. Findings from the Treatment Options for Type 2 Diabetes study and the Restoring Insulin Secretion Pediatric Medication Study illustrate the aggressive nature of T2D in youth and assert the urgent need for efficacious diabetes prevention strategies for at-risk youth [8,9].

Diabetes prevention strategies for adults have seen significant progress [10-17], but less is known about effective diabetes prevention strategies for adolescents. The Bright Bodies Healthy Lifestyle program and The Every Little Step Counts-Diabetes Prevention Program show promising outcomes for modifying risk factors for developing T2D in adolescents [18,19]. However, behavior modification of adolescents is complex as family dynamics at home play a pivotal role in facilitating change and shaping attitudes and beliefs about food choices and physical activity [20-26]. Low socioeconomic status, limited access to healthy food choices and physical activity, and individual motivators pose further barriers to lifestyle changes in high-risk adolescents [27-33]. These complexities make it especially important to solicit the expertise of adolescents and their families in shaping prevention strategies [34]. This can be accomplished by using human-centered design (HCD) techniques.

HCD is a qualitative problem-solving approach that engages stakeholders in the process of exploration, development, and implementation of solutions [35]. HCD is particularly effective in facilitating multidisciplinary collaboration, eliciting deep insights, and creating solutions compatible with end stakeholders’ needs [36]. HCD may be a promising approach for diabetes prevention [37], but more studies on the application of HCD in health care are needed [38].

For these reasons, we engaged at-risk adolescents, their parents, and professionals in a series of HCD sessions to answer the following questions: (1) what environmental factors inhibit/promote lifestyle changes in adolescents and their families? (2) what are the ideal characteristics of a diabetes prevention program for this population? and (3) what are the effective strategies to engage adolescents and their families in a diabetes prevention program? This paper describes the first phase of a study to co-design a diabetes prevention program for adolescents and their parents by using HCD.

## Methods

### Study Design

This formative study consisted of 4 HCD sessions: 1 professional session, 2 adolescent and parent sessions, and 1 adolescent-only session. We collaborated with Research Jam, which is part of the Indiana Clinical and Translational Sciences Institute’s Patient Engagement Core [39]. Research Jam is a multi-disciplinary team of bachelor’s and master’s level human-centered designers and health services researchers with experience in using HCD methods to engage stakeholders in exploring health challenges and cocreating solutions.

### Participant Recruitment

#### Professionals

We recruited a group of individuals with expertise in diabetes prevention or adolescents (referred to as professionals for brevity) to participate in 1 HCD session. Initially, the primary investigator reached out to colleagues who were involved in diabetes prevention or experts working with adolescents. Examples include physicians, diabetes educators, and community youth organizations. Additional professionals were recruited through a snowball sampling technique as initial participants invited colleagues who met the aforementioned criteria. Professionals were invited to participate in one 90-minute session and were compensated with US $50 per hour for their time.

#### Adolescents and Parents

Adolescents and parents were recruited in 4 ways. First, the professional group members recruited adolescents and parents in connection with their organizations by distributing flyers and word of mouth. Second, adolescents and parents were recruited from a youth diabetes prevention clinic. Third, adolescents and parents were recruited from an existing local family-focused nutrition and physical activity program. Finally, a school-based adolescent group was recruited by a school nurse from a local high school.

### Inclusion Criteria

The inclusion criteria for adolescents were (1) between the ages of 10 years and 17 years, (2) overweight (BMI ≥85th percentile for age and gender, weight for height ≥85th percentile, or weight ≥120% of 50th percentile for height), (3) with 2 additional risk factors for T2D (diagnosis of prediabetes, family history of T2D in first-degree or second-degree relatives, belong to racial/ethnic minority group with high risk, have conditions associated with insulin resistance, have had gestational diabetes or exposure to gestational diabetes in utero), (4) English speaking, and (5) a parent willing to participate in the family session (with the exception of the high school group).

Two adolescent and parent research sessions were offered simultaneously in the same building but in separate rooms. The school-based adolescent group took part in 1 session at a high school. Participants were compensated with US $20 per hour of their time. These sessions lasted 3 hours. The Indiana University Institutional Review Board approved this study and participants provided written informed consent prior to engaging in any research activities. In order to address ethical considerations, adolescents younger than 14 years underwent the assenting process, documents were written at a sixth-grade reading level, and compensation was set at a level that covers participants’ time and effort without being coercive.

### Data Collection

Research Jam facilitated the HCD sessions. All sessions were audio recorded with participant permission. Research Jam recorded field notes during activities and discussions. Each session consisted of activities that aligned with the study objectives to (1) better understand environmental factors that inhibit/promote lifestyle changes, (2) elucidate desired program characteristics, and (3) explore improved activation in diabetes prevention programs (Table 1). The purpose of the activities was to understand diabetes prevention from the participants’ perspective and elicit desired prevention strategies. Activities used a variety of methods to explore participant insights, including barrier mapping, envisioning and enacting, drawings, and discussions. For instance, Research Jam utilized a drawing activity as a method to elicit tacit knowledge. Small groups of participants were instructed to draw the “worst diabetes prevention program ever” and share their drawings with the larger group for discussion. Another activity involved drawing the “best party ever,” and then modifying the party to motivate healthy behaviors. The outcomes of the drawing activities acted as a catalyst for brainstorming among participants. To engage participants in a potentially sensitive topic, Research Jam used activities that were age appropriate and nonjudgmental. Adolescent and parent sessions began with a Forever/Never icebreaker. Participants shared something they wished to do all the time and something they wished to never do again. This allowed group members to get to know each other while providing information about the possible components to include in the program design (Multimedia Appendix 1).

**Table 1:** Objectives of the sessions and the activities performed.

Sessions, activities			Objective
**Adolescents and parents session 1**			
	Perception of risk	Engagement strategies	
	Forever/Never icebreaker	Ideal characteristics	
	Barrier issue posters (Part 1)	Environmental factors	
	Barrier issue posters (Part 2)	Ideal characteristics	
	Worst/Best program ever drawing	Ideal characteristics and engagement strategies	
	Program pitch	Ideal characteristics and engagement strategies	
**Adolescents and parents session 2**			
	Barrier issue posters (response)	Environmental factors and ideal characteristics	
	Forever/Never icebreaker	Ideal characteristics	
	Motivator discussion	Engagement strategies	
	Worst/Best program ever drawing	Ideal characteristics and engagement strategies	
**School-based adolescent session**			
	Cartoon caption	Engagement strategies	
	Forever/Never icebreaker	Ideal characteristics	
	Diabetes prevention party drawing	Ideal characteristics and engagement strategies	
**Professionals session**			
	Barrier mapping	Ideal characteristics and environmental factors	
	Best program ever drawing	Ideal characteristics and engagement strategies	
	Bad idea parking lot	Ideal characteristics	

### Analysis

The Research Jam project lead conducted collaborative analysis meetings with 4 members of Research Jam who helped facilitate the sessions. The team physically separated individual pieces of explicit data onto slips of paper. Data that required interpretation such as drawings were analyzed by capturing components included in the drawings onto slips of paper. For example, a picture of people sitting still in chairs was coded as physically inactive. This work was reviewed by Research Jam team members to ensure all data were captured. The team then used affinity mapping to physically organize the data pieces by similarity [40]. Team members continuously discussed data groupings and theme identification to iteratively refine and ensure consensus. This was particularly important as Research Jam staff members facilitated different sessions and no member was involved in every session. Next, Research Jam staff members mapped the themes related to the ideal diabetes program and engagement strategies by population to the Activity, Environment, Interaction, Object and User framework, which is based on ethnography traditions and data organization into activities (goal-directed sets of actions), environments (the arena in which activities take place), interactions (interplays between people or objects), objects (key elements that make up the environment or with which people may interact), or users (people active in the environment) categories [41] (Multimedia Appendix 2). Research Jam did not use the user category as the participants did not discuss the ideal program users.

## Results

### Session Participation

#### Professionals

Fourteen individuals participated in the professional group session. This session took place at a community church that had space to accommodate this group size. The group consisted of physicians, researchers, diabetes educators, school personnel, Young Men’s Christian Association staff members, a nurse manager at a community health center, a church pastor, a youth mentor, and a youth counselor.

#### Adolescents and Parents

The first adolescent and parent session consisted of 18 people with 5 parents and 13 adolescents and took place at a community church. The second adolescent and parent session consisted of 14 people with 6 parents and 8 adolescents and took place at a community center. The school-based adolescent group session consisted of 12 adolescents and took place at a high school.

#### Session Findings

The session findings are presented based upon the objectives of the session. No other evaluation of participation was conducted.

### Environmental Factors That Promote Adopting Lifestyle Changes

#### Healthy Choices Are Acceptable

Both adolescents and professionals stated that creating an environment where healthy choices are acceptable and appealing was important. One professional stated, “What they would actually want to do, not what they feel like they’re supposed to do or have to do, but that the healthy choice is like the awesome choice.”

#### Affirming

Both adolescents and professionals stated that it was important that a diabetes prevention program was affirming and not judgmental.

#### Focus on Positive

Parents and adolescents reported they wanted a program that avoided focusing on what not to do. This was best illustrated with cupcakes on the table with a sign reading “do not eat” (Multimedia Appendix 3).

#### Try New Things

Adolescents and parents felt that trying new foods and activities was an important part of adopting healthy behaviors that fit their lifestyle. Adolescents were interested in trying activities that were out of the ordinary or taking ordinary things and experiencing them in novel ways. For instance, adolescents created a drinkable swimming pool as a component of the diabetes prevention party (Multimedia Appendix 4). This idea represents a new spin on the recommendation to drink water instead of sugary drinks.

### Barriers to Adopting Lifestyle Changes

#### Environmental Barriers

Adolescents, parents, and professionals reported barriers such as lack of access to reliable transportation, access to healthy foods, and safe places for physical activity.

#### Limited by Availability

Adolescents reported that food choices are often limited by the options offered by parents, schools, and vending machines. As one adolescent described, “If you go to the vending machine, everything has some sort of sugar or fat in it. There aren’t really options for healthy stuff.”

#### Cost of Healthy Foods

All groups discussed the costs of healthy versus processed foods as a significant barrier. In addition, participants viewed wasted food as wasted money and viewed free food as free money such as buffets and free refills.

#### Lack of Time

Parents felt that the time required to cook healthy meals and engage in exercise was a luxury not afforded to them. As one parent explained, “When they don’t get home and mom doesn’t get home until late, then it’s like, okay throw a pizza in.”

#### Competing Priorities

All participant groups identified competing priorities as a barrier. The demands of work, school, financial strain, and unsafe neighborhoods made healthy lifestyle change a lower priority than imminent needs. Adolescents felt that a long sedentary school day coupled with required evening homework time impeded efforts to increase physical activity. Parents’ comments regarding the workday and challenges incorporating physical activity mirrored the adolescents’ sentiments on this topic. Additionally, parents verbalized safety concerns around independent outdoor play for youth. One parent described how different her childhood was from that of her children, “Even though I tried, my kids’ life is so much different from the way I was raised. Some of it was because I was afraid to send them out to play—but I was out and my mom didn’t know where I was most of the day…I would ride my bike 2 or 3 miles from home. That’s just not my kids’ existence. They’ve never had that.”

#### Food is Addictive

Adolescents and parents described foods and beverages high in sugar or salt as having an addictive quality. As one parent stated, “I’m trying desperately to find things that taste good and are healthy.” They also felt that situations where others continued to eat those foods in their presence hindered their efforts. Adolescents specifically called out celebrations, which so often center around unhealthy foods, as problematic for maintaining healthy eating habits.

### Ideal Characteristics of a Diabetes Prevention Program for Adolescents and Their Families

#### Fun

All groups reported that having fun should be the primary focus of a diabetes prevention program.

#### Importance of Play

Adolescents, parents, and professionals identified “play” as an important factor in an ideal program. Adolescents expressed play as participation in sports and free movement activities (eg, basketball, swimming, volleyball, dancing). Parents identified play as hands-on learning activities such as cooking classes, recipe sharing, and exercises not available at home.

#### No Lectures

All participant groups were averse to didactic lecture-style sessions. For instance, the worst program ever drawings included an instructor in front of a class saying “blah, blah, blah” and a participant saying, “May I speak now?” (Multimedia Appendix 3). Additionally, an adolescent stated, “A lecture is when someone talks at you instead of talking with you.” Participants wanted hands-on, collaborative, and motivating learning experiences such as cooking and socializing. All participants wanted to avoid homework and handouts. As one parent stated, “I want to know how to cook the way my mother cooked but substitute things that are healthier, so I can still get the foods that I like.”

#### Facilitator Characteristics

Adolescents wanted a facilitator who incorporates fun. Parents desired a facilitator that adolescents can look up to. All participants wanted program staff who were fun, engaging, and respectful.

#### Rewarding Success

Adolescents, parents, and professionals saw value in rewarding success. Adolescents verbalized money or access to an experience as motivating rewards. For instance, in one of the diabetes prevention party drawings, adolescents envisioned a reward where trying healthy behaviors gained them access to “the real party” (Multimedia Appendix 4). Parents verbalized rewards such as gym memberships while professionals thought that free or discounted food or cooking equipment were good incentives.

#### Try New Foods

A central focus for all participants was the inclusion of delicious healthy foods to try.

#### Build Relationships

Adolescents, parents, and professionals saw value in friendships and personal relationships as part of the program. All participants identified personal relationships as vital to the success of their program or party drawings. One adolescent explained, “I think you get to know the people that you’re going to be doing the class with and it’s a lot easier. So, if you do some sort of like fun game or activity at the beginning and people get to know each other pretty well, it’s much easier to have a good time.” The ability to collaborate was also important in their drawings.

### Effective Strategies to Engage Adolescents and Their Families

#### Use the Right Messaging

All participants agreed that messaging can be vital to getting adolescents to the program. Parents suggested using an acronym that sounded fun and social, such as “FIT: Fight it Together.” Adolescents suggested using messaging that sounded better than the actual program in order to get adolescents to the door. One adolescent answered the question about how to get adolescents to attend a diabetes prevention program by saying, “Probably make it sound better than it is because once they are there, they probably won’t leave.”

#### Reward Healthy Behaviors

All groups thought it was important to use rewards that promote healthy behaviors and celebrate success.

#### Use Inviting Language

Adolescents felt that “diabetes prevention” and “health” were not motivating messages to lead with because they were associated with uninteresting didactic learning experiences such as health class. Components of the program that adolescents and parents found most important (eg, play, making friends, trying new things, being active) should be highlighted in visual and written messaging and marketing of the program.

## Discussion

There is a need for efficacious diabetes prevention interventions for adolescents and their parents. We used HCD methods to better understand barriers, improve diabetes prevention program design, and optimize participant engagement. We found that lack of financial resources, limited access to healthy foods and safe places for physical activity, and competing priorities were significant barriers to adopting lifestyle changes. This is consistent with the findings that adolescents of low socioeconomic status have lower quality diets and lower levels of physical activity than their counterparts of high socioeconomic status [30].

We found that participants want interactive, novel, hands-on learning sessions that incorporate a sense of fun and play. Adolescents and their parents desire opportunities to try new behaviors in a supportive group environment and to work toward healthy incentives and rewards. It is important to make healthy choices intrinsically motivating because they are fun, they align with important values, or they are part of someone’s identity. If healthy choices are seen as obligatory, boring/uncool, or unenjoyable, they are less likely to be adopted. Stakeholders are averse to “one-size-fits-all” lecture-style sessions that focus on “what not to do,” recommending that the focus be kept on their interests.

Other types of formative research have been used in diabetes prevention program design. Vangeepuram and colleagues [42] conducted in-depth interviews with youth workers and focus groups with adolescents to learn about program preferences. They found that offering choices, interactive workshops, personal stories, and games were the preferred methods for program delivery [42]. Community-based participatory research, another collaborative approach to research design, has been shown to be feasible and effective with adolescents in the design of health interventions [43-47]. MacDonald et al [47] used arts-based methods to engage adolescents in the process of designing a sexual health curriculum and concluded that partnering with adolescents improved the relevance of a prevention resource for them. Unfortunately, the inclusion of adolescents and parents in the design of prevention messaging and curriculum is often neglected [34]. Traditional pediatric weight management approaches often used to decrease T2D risk focus on evidenced-based lifestyle changes to promote healthy weight [48]. While evidenced-based messaging is key, program design is not typically informed by adolescents, their parents, or the professionals in their community. This may contribute to poor outcomes and attrition [49-51]. In this study, we engaged not only adolescents but also parents and professionals in the design of a diabetes prevention program that they would want to use. Thus, this study addresses this gap in the literature by describing HCD methods and findings to better understand barriers, design diabetes prevention programs, and activate at-risk adolescents and their families.

This study has the following limitations. Recruitment strategies may have attracted participants who were more keen on making lifestyle changes. Participant perspectives may not be representative of the general population as the sample was small and from 1 urban community. Demographics of the research participants were not collected. The HCD sessions focused on desired program content rather than the delivery platform. We plan to further translate these findings into a curriculum and test its effectiveness in a larger sample size. Future research should investigate participant engagement by using different delivery modalities.

The findings of this study highlight important insights regarding diabetes prevention and lifestyle change from 3 specific stakeholder groups and demonstrate that, with appropriate methods and facilitation, adolescents, parents, and professionals can be empowered to co-design diabetes prevention programs.

## Appendix

Multimedia Appendix 1 Human-centered design activities.

Multimedia Appendix 2 Activity venn diagram.

Multimedia Appendix 3 Drawing of the "worst program ever".

Multimedia Appendix 4 Diabetes prevention party.

## Abbreviations

HCD: human-centered design
T2D: type 2 diabetes

## References

[1] Skinner AC, Ravanbakht SN, Skelton JA, Perrin EM, Armstrong SC (2018). Prevalence of Obesity and Severe Obesity in US Children, 1999-2016. Pediatrics.

[2] Li C, Ford ES, Zhao G, Mokdad AH (2009). Prevalence of pre-diabetes and its association with clustering of cardiometabolic risk factors and hyperinsulinemia among U.S. adolescents: National Health and Nutrition Examination Survey 2005-2006. Diabetes Care.

[3] Mayer-Davis EJ, Lawrence JM, Dabelea D, Divers J, Isom S, Dolan L, Imperatore G, Linder B, Marcovina S, Pettitt DJ, Pihoker C, Saydah S, Wagenknecht L, SEARCH for Diabetes in Youth Study (2017). Incidence Trends of Type 1 and Type 2 Diabetes among Youths, 2002-2012. N Engl J Med.

[4] Imperatore G, Boyle JP, Thompson TJ, Case D, Dabelea D, Hamman RF, Lawrence JM, Liese AD, Liu LL, Mayer-Davis EJ, Rodriguez BL, Standiford D, SEARCH for Diabetes in Youth Study Group (2012). Diabetes Care.

[5] Copeland KC, Zeitler P, Geffner M, Guandalini C, Higgins J, Hirst K, Kaufman FR, Linder B, Marcovina S, McGuigan P, Pyle L, Tamborlane W, Willi S, TODAY Study Group (2011). Characteristics of adolescents and youth with recent-onset type 2 diabetes: the TODAY cohort at baseline. J Clin Endocrinol Metab.

[6] Pettitt DJ, Talton J, Dabelea D, Divers J, Imperatore G, Lawrence JM, Liese AD, Linder B, Mayer-Davis EJ, Pihoker C, Saydah SH, Standiford DA, Hamman RF, SEARCH for Diabetes in Youth Study Group (2014). Prevalence of diabetes in U.S. youth in 2009: the SEARCH for diabetes in youth study. Diabetes Care.

[7] Rhodes ET, Prosser LA, Hoerger TJ, Lieu T, Ludwig DS, Laffel LM (2012). Estimated morbidity and mortality in adolescents and young adults diagnosed with Type 2 diabetes mellitus. Diabet Med.

[8] Zeitler P, Hirst K, Pyle L, Linder B, Copeland K, Arslanian S, Cuttler L, Nathan DM, Tollefsen S, Wilfley D, Kaufman F, TODAY Study Group (2012). A clinical trial to maintain glycemic control in youth with type 2 diabetes. N Engl J Med.

[9] RISE Consortium (2018). Impact of Insulin and Metformin Versus Metformin Alone on β-Cell Function in Youth With Impaired Glucose Tolerance or Recently Diagnosed Type 2 Diabetes. Diabetes Care.

[10] Absetz P, Valve R, Oldenburg B, Heinonen Heikki, Nissinen Aulikki, Fogelholm Mikael, Ilvesmäki Vesa, Talja Martti, Uutela Antti (2007). Type 2 diabetes prevention in the "real world": one-year results of the GOAL Implementation Trial. Diabetes Care.

[11] Ackermann RT, Finch EA, Brizendine E, Zhou H, Marrero DG (2008). Translating the Diabetes Prevention Program into the community. The DEPLOY Pilot Study. Am J Prev Med.

[12] Amundson HA, Butcher MK, Gohdes D, Hall TO, Harwell TS, Helgerson SD, Vanderwood KK, Montana Cardiovascular DiseaseDiabetes Prevention Program Workgroup (2009). Translating the diabetes prevention program into practice in the general community: findings from the Montana Cardiovascular Disease and Diabetes Prevention Program. Diabetes Educ.

[13] Boltri JM, Davis-Smith YM, Seale JP, Shellenberger S, Okosun IS, Cornelius ME (2008). Diabetes prevention in a faith-based setting: results of translational research. J Public Health Manag Pract.

[14] Kanaya AM, Santoyo-Olsson J, Gregorich S, Grossman M, Moore T, Stewart AL (2012). The Live Well, Be Well study: a community-based, translational lifestyle program to lower diabetes risk factors in ethnic minority and lower-socioeconomic status adults. Am J Public Health.

[15] Katula JA, Vitolins MZ, Rosenberger EL, Blackwell CS, Morgan TM, Lawlor MS, Goff DC (2011). One-year results of a community-based translation of the Diabetes Prevention Program: Healthy-Living Partnerships to Prevent Diabetes (HELP PD) Project. Diabetes Care.

[16] Knowler WC, Barrett-Connor E, Fowler SE, Hamman RF, Lachin JM, Walker EA, Nathan DM, Diabetes Prevention Program Research Group (2002). Reduction in the incidence of type 2 diabetes with lifestyle intervention or metformin. N Engl J Med.

[17] McTigue KM, Conroy MB, Bigi L, Murphy C, McNeil M (2009). Weight loss through living well: translating an effective lifestyle intervention into clinical practice. Diabetes Educ.

[18] Savoye M, Caprio S, Dziura J, Camp A, Germain G, Summers C, Li F, Shaw M, Nowicka P, Kursawe R, Depourcq F, Kim G, Tamborlane WV (2014). Reversal of early abnormalities in glucose metabolism in obese youth: results of an intensive lifestyle randomized controlled trial. Diabetes Care.

[19] Shaibi GQ, Konopken Y, Hoppin E, Keller CS, Ortega R, Castro FG (2012). Effects of a culturally grounded community-based diabetes prevention program for obese Latino adolescents. Diabetes Educ.

[20] Berge JM (2009). A review of familial correlates of child and adolescent obesity: what has the 21st century taught us so far?. Int J Adolesc Med Health.

[21] Epstein LH, Wisniewski L, Weng R (1994). Child and parent psychological problems influence child weight control. Obes Res.

[22] Golan M (2006). Parents as agents of change in childhood obesity--from research to practice. Int J Pediatr Obes.

[23] Golan M, Weizman A (2001). Familial Approach To The Treatment Of Childhood Obesity: Conceptual Model. Journal of Nutrition Education.

[24] Golan M, Crow S (2004). Targeting parents exclusively in the treatment of childhood obesity: long-term results. Obes Res.

[25] Ritchie LD, Welk G, Styne D, Gerstein DE, Crawford PB (2005). Family environment and pediatric overweight: what is a parent to do?. J Am Diet Assoc.

[26] Stein RI, Epstein LH, Raynor HA, Kilanowski CK, Paluch RA (2005). The influence of parenting change on pediatric weight control. Obes Res.

[27] Alm M, Soroudi N, Wylie-Rosett J, Isasi CR, Suchday S, Rieder J, Khan U (2008). A qualitative assessment of barriers and facilitators to achieving behavior goals among obese inner-city adolescents in a weight management program. Diabetes Educ.

[28] Burdette HL, Whitaker RC (2004). Neighborhood playgrounds, fast food restaurants, and crime: relationships to overweight in low-income preschool children. Prev Med.

[29] Estabrooks PA, Lee RE, Gyurcsik NC (2003). Resources for physical activity participation: does availability and accessibility differ by neighborhood socioeconomic status?. Ann Behav Med.

[30] Hanson MD, Chen E (2007). Socioeconomic status and health behaviors in adolescence: a review of the literature. J Behav Med.

[31] Larson NI, Story MT, Nelson MC (2009). Neighborhood environments: disparities in access to healthy foods in the U.S. Am J Prev Med.

[32] Rossen LM, Talih M (2014). Social determinants of disparities in weight among US children and adolescents. Ann Epidemiol.

[33] Weir LA, Etelson D, Brand DA (2006). Parents' perceptions of neighborhood safety and children's physical activity. Prev Med.

[34] Santelli JS, Smith Rogers Audrey, Rosenfeld WD, DuRant RH, Dubler N, Morreale M, English A, Lyss S, Wimberly Y, Schissel A, Society for Adolescent Medicine (2003). Guidelines for adolescent health research. A position paper of the Society for Adolescent Medicine. J Adolesc Health.

[35] Chen E, Leos C, Kowitt SD, Moracco KE (2020). Enhancing Community-Based Participatory Research Through Human-Centered Design Strategies. Health Promot Pract.

[36] Hassi L, Laakso M, Karjalainen TM, Koria M, Salimaki M (2011). Making sense of design thinking. IDBM papers.

[37] Matheson GO, Pacione C, Shultz RK, Klügl Martin (2015). Leveraging human-centered design in chronic disease prevention. Am J Prev Med.

[38] Bazzano AN, Martin J, Hicks E, Faughnan M, Murphy L (2017). Human-centred design in global health: A scoping review of applications and contexts. PLoS One.

[39] What is Research Jam.

[40] Kolko J (2011). Exposing the Magic of Design: A Practitioner’s Guide to the Methods & Theory of Synthesis.

[41] Martin B, Hanington B (2012). Universal methods of design ways to research complex problems, develop innovative ideas, and design effective solutions.

[42] Vangeepuram N, Williams N, Constable J, Waldman L, Lopez-Belin P, Phelps-Waldropt L, Horowitz CR (2017). TEEN HEED: Design of a clinical-community youth diabetes prevention intervention. Contemp Clin Trials.

[43] Bardwell G, Morton C, Chester A, Pancoska P, Buch S, Cecchetti A, Vecchio M, Paulsen S, Groark S, Branch RA (2009). Feasibility of adolescents to conduct community-based participatory research on obesity and diabetes in rural Appalachia. Clin Transl Sci.

[44] Berge JM, Mendenhall TJ, Doherty WJ (2009). Using Community-based Participatory Research (CBPR) To Target Health Disparities in Families. Fam Relat.

[45] Branch R, Chester A (2009). Community-based participatory clinical research in obesity by adolescents: pipeline for researchers of the future. Clin Transl Sci.

[46] Rogers EA, Fine SC, Handley MA, Davis HB, Kass J, Schillinger D (2017). Engaging Minority Youth in Diabetes Prevention Efforts Through a Participatory, Spoken-Word Social Marketing Campaign. Am J Health Promot.

[47] MacDonald J, Gagnon A, Mitchell C, Di Meglio Giuseppina, Rennick J, Cox J (2011). Include them and they will tell you: learnings from a participatory process with youth. Qual Health Res.

[48] Barlow SE, Expert Committee (2007). Expert committee recommendations regarding the prevention, assessment, and treatment of child and adolescent overweight and obesity: summary report. Pediatrics.

[49] Hampl S, Paves H, Laubscher K, Eneli I (2011). Patient engagement and attrition in pediatric obesity clinics and programs: results and recommendations. Pediatrics.

[50] Sallinen Gaffka BJ, Frank M, Hampl S, Santos M, Rhodes ET (2013). Parents and pediatric weight management attrition: experiences and recommendations. Child Obes.

[51] Kwitowski M, Bean MK, Mazzeo SE (2017). An exploration of factors influencing attrition from a pediatric weight management intervention. Obes Res Clin Pract.

