# Supplementary material for: Diabetes Prevention in Adolescents: Co-design Study Using Human-Centered Design Methodologies
Source: J Particip Med. 2021 Feb 24;13(1):e18245. doi: 10.2196/18245 (PMC7946580; doi:10.2196/18245)
Supplement: Multimedia Appendix 1 [file jopm_v13i1e18245_app1.docx]

Appendix

HCD Activity Detail

| Perception of Risk  Session(s):  Adolescents and parents | RJ first explored participants’ knowledge and perceptions of diabetes risk by asking adolescents and parents to write three things that increase risk for T2D. This information was used to assess knowledge and ensure messaging would effectively counter mis-information potential program participants might have. |
| --- | --- |
| Forever/Never Ice-breaker  Session(s):  Adolescents and parents  Adolescents-only | Participants were asked to share something they wished to do all the time and something they wished to never do again. This provided a quick warm-up and insights on activities to be included or avoided in program design. |
| Barrier Issue Posters  Session(s):  Adolescents and parents | Part One:  Healthy behaviors typically recommended to reduce risk for T2D (i.e. avoid sugary drinks, eat more fruits and vegetables, increase physical activity, use portion control, decrease junk foods) were presented to the group on posters. Participants were asked to independently consider each behavior and write down perceived barriers on the posters using a particular marker color. The facilitator read barriers aloud to the group to promote further discussion and understanding.  Part Two:  Participants brainstormed strategies to make each behavior more achievable. They were then instructed to consider how their solutions could resemble the “forever” activities that were verbalized during the Forever/Never Icebreaker. These ideas were written directly on the posters in a second marker color.  Response: These barrier posters were brought into a subsequent session and participants already engaged in a wellness program were asked to provide suggestions for how to overcome these barriers. |
| Worst/Best Program Ever Drawing  Session(s):  Adolescents and parents | Drawing is a method to elicit tacit knowledge, or what people know, feel, or imagine, but which “can’t be readily expressed in words” [1]. Small groups of participants were instructed to draw the “worst diabetes prevention program ever” and/or the “best diabetes prevention program ever” and share their drawings with the larger group for discussion. The goal of this activity was to elicit insights into what adolescents, parents and community and health professionals desire in a program. |
| Program Pitch  Session(s):  Adolescents and parents | This activity instructed parents and adolescents to convince the person sitting next to them to attend their ideal diabetes prevention program. This technique is a form of participatory envisioning and enacting [2] which asks participants to act out or pretend in order to, in this case, both understand and generate ideal messaging for a diabetes prevention program. As the activity progressed, certain words were banned (i.e. convince your neighbor without using the word “health” or “diabetes prevention”) in order to uncover other potential words that might be more engaging. This activity elicited messages that adolescents and parents would use to implore others to engage in a diabetes prevention program. |
| Barrier Mapping  Session(s):  Professionals | This activity was utilized in the session with community professionals. They were first asked to individually identify one outcome “they wished families would gain from participating in a diabetes prevention program.” Participants were then organized into groups of 3 to 4 and instructed to select one of the outcomes. They were given a large flipchart page divided into four sections (personal, professional, political, and cultural) and asked to write in each section barriers to their own ability to support families in achieving the selected goal. This was a dedicated flipchart where participants could write ideas they thought would be bad ideas for a program for adolescents and families. This way, these ideas could be recorded but participants could focus on ideas that might work rather than getting stuck in what currently isn’t working. This was utilized during the community professional session during the Best Program Ever Drawing Activity. |
| Motivator Discussion  Session(s):  Adolescents and Parents | The subset of adolescents and their families who were engaged in a wellness program were asked to share and discuss their motivators for attending the program each week. |
| Cartoon Caption  Session(s):  Adolescents only | This activity was used with the High School Student group to better understand the types of messages that adolescents receive from adults regarding decreasing risk for T2D and explore how they feel about these messages. This activity is a form of a probe in which participants are given a pre-designed situation to elicit a response [3]. In this case, the adolescents were given a worksheet that featured a cartoon drawing of an adult and an adolescent with speech and thought bubbles. They were asked to think of a past conversation with an adult about health. They wrote the adult’s comments in the word bubble and then captured their own verbal response in the adolescent speech bubble as well as their thoughts or feelings in the adolescent thought bubble. |
| Diabetes Prevention Party Drawing  Session(s):  Adolescents-only | Small groups of adolescents were instructed to draw their idea for the “best party ever.” After 10 minutes groups were assigned two healthy behaviors and were asked to adapt their party in such a way that would motivate guests to adopt the healthy behaviors. Groups then presented their drawings and discussed how they incorporated the healthy behaviors. Beginning with a party avoided pre-conceived notions of diabetes prevention or health programming in their ultimate program design. |

**References**

1. Sanders EBN. Useful and Critical: The Position of Research in Design. Paper presented at: Postdesign and Participatory Culture September 1999, 1999; Tuusula, Finland.
2. Sanders E, Stappers P. Probes, toolkits and prototypes: Three approaches to making in codesigning. *CoDesign.* 2014;10.
3. Sanders E, Brandt E, Binder T. A framework for organizing the tools and techniques of participatory design. Proceedings of the 11th Biennial Participatory Design Conference; 2010; Sydney, Australia
