# Supplementary figures and images for: Diabetes Prevention in Adolescents: Co-design Study Using Human-Centered Design Methodologies
Source: J Particip Med. 2021 Feb 24;13(1):e18245. doi: 10.2196/18245 (PMC7946580; doi:10.2196/18245)

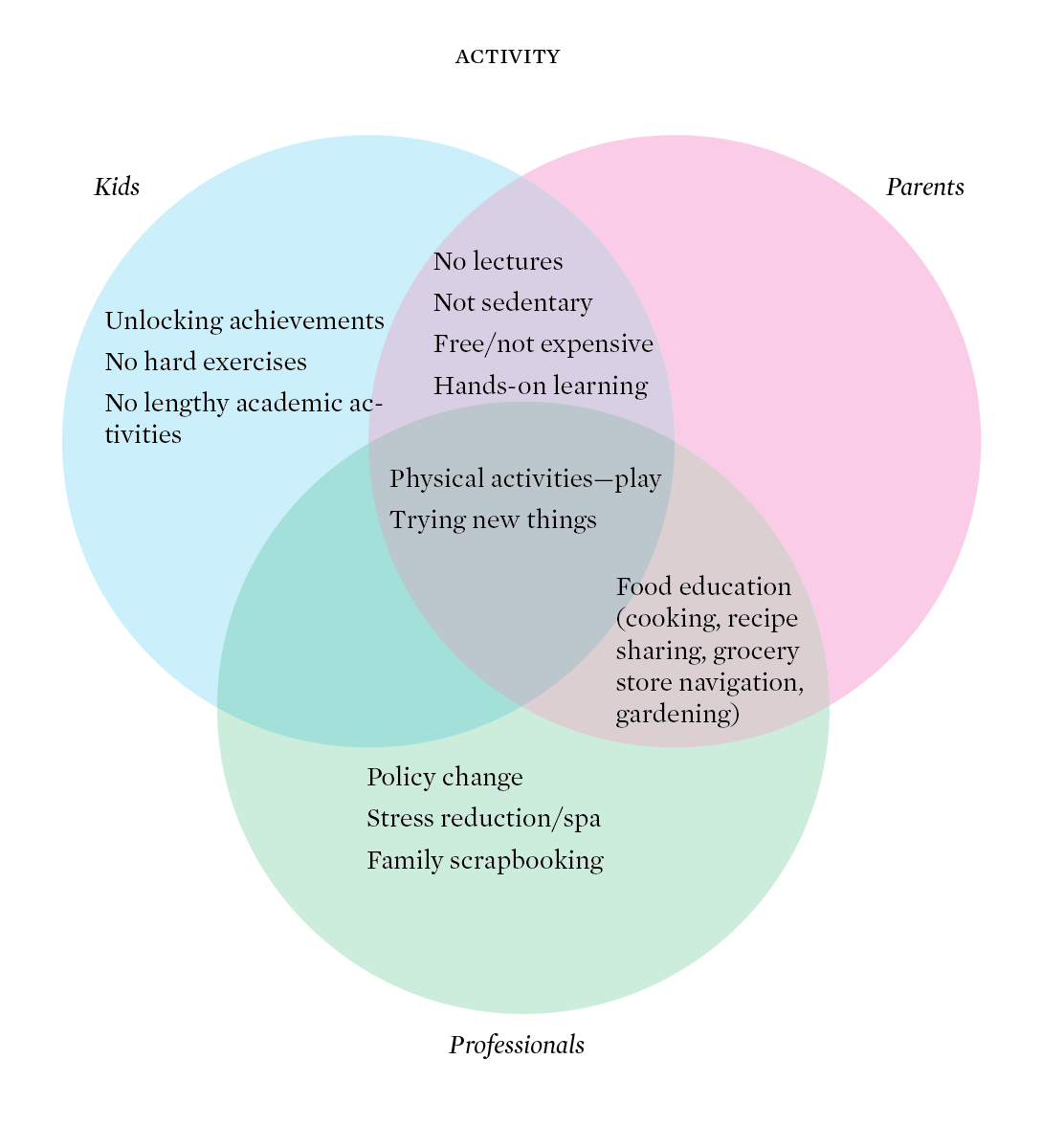

Supplement: Multimedia Appendix 2 [file jopm_v13i1e18245_app2.png]

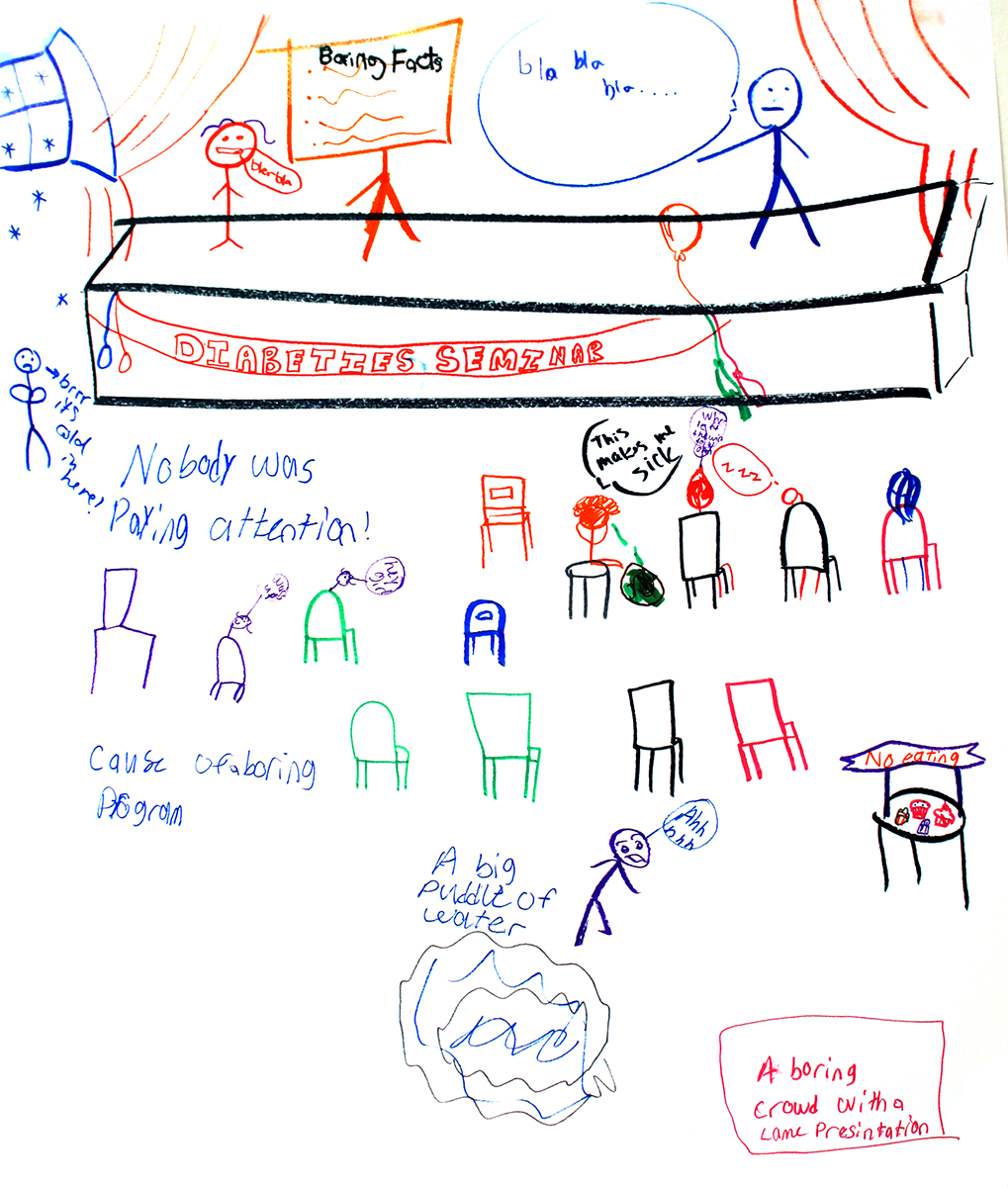

Supplement: Multimedia Appendix 3 [file jopm_v13i1e18245_app3.png]

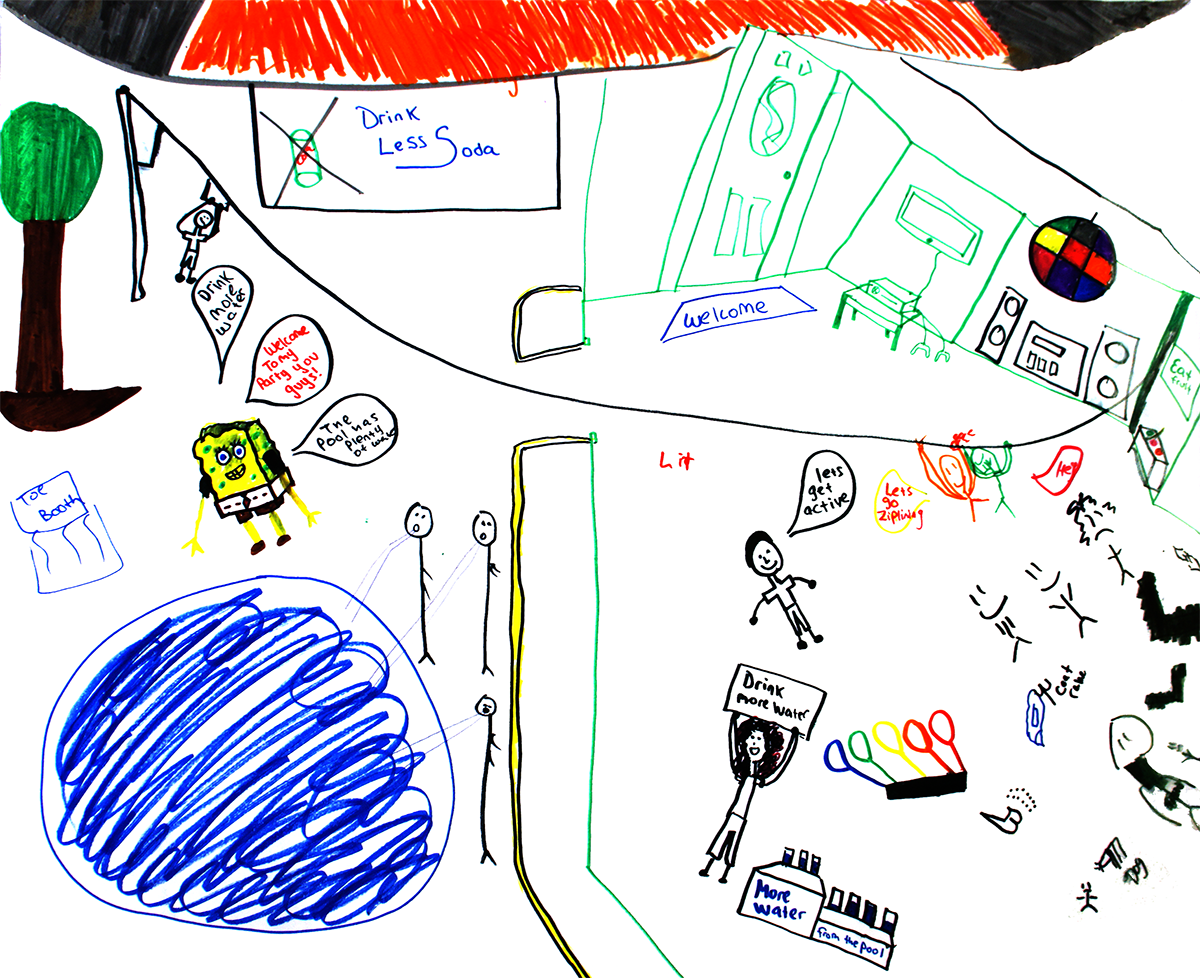

Supplement: Multimedia Appendix 4 [file jopm_v13i1e18245_app4.png]
